# Microbiota in the Gastrointestinal Tract

**DOI:** 10.3390/medsci6040116

**Published:** 2018-12-14

**Authors:** Walburga Dieterich, Monic Schink, Yurdagül Zopf

**Affiliations:** 1 Medical Clinic 1, Friedrich-Alexander-Universität Erlangen-Nürnberg, Ulmenweg 18, 91054 Erlangen, Germany; Monic.schink@uk-erlangen.de (M.S.); yurdaguel.zopf@uk-erlangen.de (Y.Z.); 2 Hector Center of Excellence for Nutrition, Exercise and Sports, University of Erlangen-Nürnberg, 91054 Erlangen, Germany

**Keywords:** microbiota, gastrointestinal tract

## Abstract

Gut microbiota are permanent residents of humans with the highest concentrations being found in human colon. Humans get the first contact with bacteria at delivery, and microbiota are subject of permanent change during the life. The individual microbiota pattern is highly variable and varying environmental conditions, e.g., diets, antigen exposure, infections, or medication, as well as genetics, age, or hygiene factors, strongly influence the bacterial community. A fine interaction between the host and microbiota determines the outcome of health or disease. The gut immune system is constantly challenged to distinguish between commensal non-invasive bacteria and potential pathogens. Goblet cells produce mucins that prevent most gut bacteria from penetrating through intestinal epithelial barrier, and Paneth cells are the main supplier of anti-microbial defensins. Gut epithelial and immune cells recognize bacteria via surface markers and they initiate an adequate immune answer. A dysbiosis is noticed in several diseases, but the crucial role in pathogenesis has to be proven. Prebiotics or probiotics are discussed as valuable tools to preserve or restore a healthy gut community.

## 1. Introduction

Bacteria are ubiquitously present in the environment, air, soil, water, and large amounts are resident on human skin and especially in the gastrointestinal tract. The form of bacteria is multifarious with coccid, rod-shaped, spirillum, or budding shapes. According to their use and tolerance against oxygen, they can be grouped into aerobic, anaerobic, or microaerophilic organisms. Bacteria are classified depending on shape, characteristics of cell membranes, and usage of energy, e.g., heterotrophic, phototrophic, or lithotrophic. Optimal conditions allow for bacteria to multiply every 20 min, resulting in a high progeny in a short time and the ability to adapt to changing environmental conditions.

For a long time the main focus has been directed at bacteria that are pathogenic for humans, e.g., *Streptococcus pyogenes*, *Bordetella pertussis*, *Corynebacterium diphtheriae*, *Clostridium tetani*, *Salmonella typhimurium*, *Vibrio cholera*, and many others. However, microbiota coexist in close association with humans and most of them are not harmful, but rather important for the host.

The microbiota have great impact on nutrient degradation and adsorption, influence defense against pathogens, stimulate the immune system, and affect gut health. A high variability of microbiota is known between individuals and the microbial pattern is influenced by genetics, age, personal hygiene, infection, medication, and diet [1,2,3].

## 2. Microbiota in the Early Years of Life

The newborn comes into contact with microorganisms at birth and bacterial communities successively develop, especially during the first months of life. At delivery, the oral and nasopharyngeal membrane as well as skin and gut of the baby colonize with microorganisms. Interestingly, no major microbial variations have been found in the different habitats from newborns (skin, oral, or nasopharyngeal mucosa, gut), which is in striking contrast to the varying microbial colonization of mothers’ different body habitats [4].

The mode of delivery itself is decisive for the first bacterial colonization. It has been shown that vaginally delivered babies have acquired microbiota that are highly similar to maternal vaginal flora, and are dominated by *Lactobacillus, Prevotella*, and *Sneathia* spp. Babies who were born by Cesarean section (C-section) mainly possess microbiota that occur naturally on skin, e.g., *Staphylococcus*, *Corynebacterium*, and *Propionibacterium* spp., and they are not very similar to maternal skin microbiota [4]. The basic microbial pattern substantially differs between babies. During the first years of life, the gut microbiota modify successively, but a distinct feature of individual gut microorganisms is maintained, and thus suggests a competitive advantage of early settled bacteria [5]. The basic colonization and bacterial diversity seem important for the development of a well-balanced interaction between microbiota and host. When reaching the end of the first 2–3 years, the microbiota stabilize and resemble the bacterial composition of the adult gut [6]. Studies have shown that babies born by Cesarean section more often suffer from wheezing and allergic sensitization during the first two years [7]. In addition, C-section has been associated with a modest increased risk for food allergy, allergic rhinitis, asthma, and intestinal bacterial infection at age 1–2 years. Nevertheless, the causality of C-section for allergic reactions or bacterial infection is marginal, and thus the mode of delivery seems to play only a minor role in the establishment of a robust microbiota [8,9].

Interestingly, microbiota of monozygotic twins are more similar to each other when compared to unrelated individuals [10]. In addition, twins showed similar sequential variations in their microbiota profile, and thus underline the importance of environmental conditions for establishing the gut flora [11]. It has been shown that infant diet strongly impacts the intestinal flora. Breast-feeding favors the bacterial diversity and richness, and intestinal colonization with *Lactobacillus*, *Staphylococcus*, *Megasphaera*, and class of Actinobacteria, whereas formula feeding causes enrichment in Clostridiales and phylum Proteobacteria [6]. Furthermore, the application of antibiotics affects the bacterial community in early babyhood, especially in the first year of life, and causes a delayed or altered colonization of gut bacteria, with the depletion of Enterobacteriaceae, Lachnospiraceae, and Erysipelotrichaceae [6].

In addition to the above-mentioned aspects (delivery mode, infant diet, antibiotics) lots of other factors might influence the complexity of gut microbiota and the adaption of bacterial communities over the years, e.g., host genetics, age, life style, hygiene factors, allergen contact, diets, consumption of pro- or prebiotics, or infections [1,12].

## 3. Microbiota Along and Across the Gut

The concentration of microbiota increases steadily along the gastrointestinal tract, with small numbers in the stomach, but very high concentrations in the colon. The stomach and proximal duodenum are exceptionally inhospitable, and very few bacteria are resistant to this acidic condition, to bile or pancreatic enzymes, and they can survive or multiply.

The stomach harbors only 10^1^ bacteria per gram content, and increasing densities and bacterial diversities are found in the duodenum (10^3^/g), jejunum (10^4^/g), ileum (10^7^/g), and colon (10^12^ bacteria/gram) [13]. Most human gut microorganisms are strictly anaerobic and they belong to phyla Bacteroidetes, Firmicutes, and Proteobacteria. Other gut bacteria with minor absolute percentage in healthy gut (usually below 1%) mostly belong to phyla Actinobacteria, Verrumicrobia, Acidobacteria, or Fusobacteria [1,14]. Mucosa-associated bacteria from distal small intestine and the colon are dominated by phyla Bacteroidetes and Firmicutes, but they display different ratios. Mucosa-associated bacteria, which have been isolated from biopsy samples, show an enrichment of *Lactobacillus* (Firmicutes)*, Veillonella* (Firmicutes), and *Helicobacter* (Proteobacteria) in proximal gut, whereas *Bacilli* (Firmicutes), Streptococcaceae (Firmicutes), Actinomycinaeae, and Corynebacteriaceae (both Actinobacteria) are abundant in duodenum, jejunum, or ileum, and increased proportions of Lachnospiraceae (Firmicutes) and Bacteroidetes are found in the colon [13,15].

In addition to longitudinal variations, the microbial patterns also differ between the gut epithelium and gut lumen. Goblet cells are the main producers of glycosylated proteins, so-called mucins that form a dense protective mucus layer and prevent most bacteria from penetration [1]. Only specialized bacteria are able to adhere to mucus [16], use the mucus as nutrient source, or get access to epithelial cells, e.g., *Clostridium*, *Lactobacillus*, or *Enterococcus*. In contrast, feces harbor numerous different species, belonging to *Bacteroides*, *Bifidobacterium*, *Streptococcus*, Enterobacteriaceae, *Enterococcus*, *Clostridium*, *Lactobacillus*, and *Ruminococcus* [13].

Most studies have paid attention to bacteria that are present in feces, but only limited information is available for mucosa-associated microorganisms. This is not surprising because the isolation of mucosa-associated bacteria is much more complex and needs the taking of intestinal biopsies during coloscopy, whereas fecal samples are easy to collect. Significant differences in bacterial composition and diversity have been shown between fecal and biopsy samples from same individual. However, the mucosal bacterial communities from ascending, transverse, and descending parts of the colon show only minor variations, and thus suggest a high stability that is caused by intensive interaction and signaling between mucosa-linked microbiota and the host [17]. Since mucosa-associated bacteria are in close contact with host epithelial cells, their influence on the immune system and gut homeostasis might be even more important, although numerically they are represented to a minor extent [18].

## 4. Viruses and Fungi

While the bacterial component of intestinal microbiota is the most common and currently the main target in microbiota study, the intestine is also colonized by archaea, bacteriophages, viruses, unicellular eukaryotes, and fungi. Although their quantitative contribution to microbial communities is minor, their functional relevance for maintaining a healthy microbial community could be significant. Recently, abnormal viral patterns have been descripted in inflammatory bowel diseases [19]. Bacteriophages, in particular, strongly impact the survival, reproduction, composition, and functionality of their bacterial hosts. A total of 23 different bacteriophages that are common in over 50% of individuals have recently been identified, and their reduced occurrence was noticed in patients with gastrointestinal complaints [20].

The human gut also harbors several fungi, including the genera Aspergillus, Candida, Cryptococcus, and Penicillium, which account for 0.2–0.3% of microbiota [18]. Although the intestinal mycobiota have not yet been related to any specific disease, alterations of fungal patterns were noticed in irritable bowel syndrome and inflammatory bowel diseases [21,22,23]. The biodiversity of fungal communities is linked to diets, e.g., Candida frequency is positively correlated with carbohydrates, Aspergillus negatively with short-chain fatty acids [18]. Since fungi are important members in food degradation and suppliers of nutritional intermediates, they deeply influence the occurrence and concentrations of metabolites and future studies should include their identification and determination.

## 5. Host—Microbiota Interaction

The gastrointestinal tract is the interface between the host and environment, and especially the small intestine has a huge surface area to control the important function of digestion and absorption of nutrients. Since the intestine is the site with the highest bacterial concentration, the host was forced to develop a strategy of tolerance to beneficial and harmless microorganisms, but also an efficient defense mechanism against pathogens and bacterial overgrowth [1].

### 5.1. Mucus Barrier

The intestine is lined by mucus, with an inner dense layer and an outer loosely adhering layer, which enlarges along the gastrointestinal tract and it is thickest in the colon. Especially the inner layer of the mucus represents a highly efficient first defense mechanism. Because of its high density, it prevents most bacteria from penetration and thus isolates the epithelium from the huge amounts of luminal microbiota. Mucins are rich in glycosylation and more than 100 different mono-, di-, or trisialylated oligosaccharides are described in human mucus. Nevertheless, the mucins show a high conservation between individuals and therefore point to an important role in the selection of commensal intestinal bacteria [16]. Only few microorganisms are able to adhere to mucus and almost exclusively to the outer mucus layer, in dependency of the possession of lectins [1,16]. Mucus-binding proteins were isolated from beneficial bacteria, e.g., *Lactobacillus reuteri*, *Lactobacillus plantarum*, and *Lactobacillus rhamnosus*. In addition, pathogenic microorganisms, like *Helicobacter pylori*, *Clostridium jejuni*, and also noroviruses, have been shown to adhere to mucus. The human histo-blood group antigens that are found on mucins are suggested as receptors [16]. Interestingly, a high abundancy of mucin-degrading *Akkermansia muciniphila* was found in healthy colon, and reduced levels were found in patients with inflammatory bowel disease. Thus, *A. muciniphila* may play an important function in maintaining intestinal integrity [24]. In this context, mice studies also revealed that bacteria itself have a great impact on formation of the mucus layer. Germ-free mice display less Goblet cells, the main producer of mucins, possess a thinner mucus layer [1], show reduced muscle wall thickness, altered cytokine and immunoglobulin levels, and are more vulnerable to infections [25].

### 5.2. Intestinal Epithelial Barrier

The intestinal epithelium separates the gut lumen from the lamina propria, and it is mainly composed of absorptive enterocytes, goblet-, Paneth-, and endoenterocrine cells. The cell layer forms a physical barrier that allows the restricted paracellular transport of molecules. The tight junction are composed of several transmembrane and cytosolic proteins, e.g., occludin, claudins, junctional-adhesion molecules, or zonula occludens, and the exact regulation of tight junction proteins is mandatory for the epithelial integrity [26]. An impaired barrier function has been associated with Crohn’s disease (CD), and inflamed mucosal sites have shown increased paracellular and vascular permeability. Interestingly, the determination of mucosal bacteria from injured sites of patients with CD show increased numbers of *Escherichia* (phylum Proteobacteria) but reduced proportions of *Lachnospira*, *Faecalibacterium*, and *Blautia*, all belonging to phylum Firmicutes, as compared to biopsies from adjacent mucosal sites without injury. The dysbiosis is suggested to favor or maintain endothelial lesions in CD [27]. Recently, a dysbiosis and invasion of bacteria has also been described in the gut of patients with ankylosing spondylitis. Ileal samples, especially from an inflamed area, show an upregulation of zonulin expression and an increased gut vascular barrier. Enhanced serum levels of zonulin alter the expression of tight junction proteins with a significant downregulation of occludin and correlate with an increased intestinal permeability in patients with ankylosing spondylitis [28].

Although animal studies further point to a crucial involvement of commensal microbiota in the formation of the colonic epithelial barrier and the maintenance of intestinal homeostasis [29], further studies must verify whether the dysbiosis is the cause or consequence of several diseases. Altogether, these data underline the well-proven interaction of microbiota and host epithelial cells and the need for fine adaptation to ongoing changing conditions.

### 5.3. Immunosensitive Cells

The gut-associated lymphoid tissue is localized all over the intestine and it ensures a tight regulation and adequate host defense to maintain the gut homeostasis. The intestinal immune system is permanently faced with lots of antigens, commensal and opportunistic microorganisms, as well as potential pathogens and thus permanently forced to discriminate between non-invasive bacteria and pathogens.

Intestinal epithelial cells and especially specialized Paneth cells that are localized at the bottom of intestinal crypts, secret anti-microbial proteins, such as lysozyme and α-/β-defensins, and during bacterial upgrowth, enterocytes and Paneth cells are activated and further secrete anti-microbial peptides, like C-type-lectin, to kill or inactivate bacteria [30].

In addition, epithelial cells and dendritic cells recognize bacteria via so called pattern recognition receptors (PPR), such as Toll-like or Nod-like receptors. Toll-like receptor 4 (TLR4) is mainly triggered by lipopolysaccharide of Gram-negative bacteria, whereas TLR2 is activated by peptidoglycan and lipoteichoic acids, the main cell wall components of Gram-positive bacteria. The PPRs display a crucial role in the activation of the immune system, and the binding of microorganisms to PPRs leads to a downstream effect with recruitment of immune cells of the native and adaptive immune system, e.g., T cells, plasma cells, macrophages, and dendritic cells, to the site of infection, and the secretion of chemokines and cytokines [3,31]. Mucosal dendritic cells are skilled for the uptake of bacteria, travel to lymph nodes, and induce an adequate immune response to commensal bacteria [3]. Studies have shown that dendritic cells are inefficient at killing pathogens, but they can keep living bacteria for several days. These bacterial-loaded dendritic cells are resident at mucosal mesenteric lymph nodes and are able to stimulate microbe-specific secretory immunoglobulin A (IgA) that inhibits the growth and penetration of bacteria [32]. From mouse studies, we also know that changes in gut microbiota cause an enrichment in numbers of IgA-secreting plasma cells going along with elevated serum IgA concentrations, and result in the protection against bacterial sepsis [33].

Commensal bacteria possess the same molecular pattern as pathogens, which are recognized by Toll-like receptors of epithelial cells or innate immune cells. However, some commensals, such as *Bacteroides fragilis,* cause a tolerogenic rather than an inflammatory response dominated by the activation of regulatory T cells and the secretion of anti-inflammatory interleukin-10 (IL-10) [31,34]. Stimulation of human peripheral blood cells with commensal gut bacteria, e.g., *Escherichia coli*, *Enterococcus,* and *B. fragilis*, which are enriched in mucosal biopsies, induce the maturation and activation of surface markers CD40 and CD83 on dendritic cells. However, each bacterium provokes an individual cytokine pattern, including the pro- and anti-inflammatory cytokines IL-23, IL-12p70, and IL-10 [35], thus demonstrating the fine tuning between the host immune system and microbiota that decides inflammation or tolerance.

### 5.4. The Gut-Brain Axis

Emerging evidence suggests an important influence of the interactions between human gut microbiota and the brain in a bidirectional manner. Whereas, gut microbiota influence the central nervous system through neuro-endocrine-, neuronal-, and immune-mediated mechanisms, the brain affects gut microbiota via the autonomic nervous system [36]. Gut microbiota are important producers of metabolites, like short-chain fatty acids, which influence the intestinal barrier function [37], the release of mucosal neurotransmitters (e.g., serontonin) [38], the sympatic nervous system [39], as well as the modulation of neurotransmitters (e.g., GABA, serotonin, acetylcholine, histamine, melatonin) [40], and the brain-derived neurotrophic factor [41]. Furthermore, gut microbiota modulate afferent sensory nerves, e.g., through the inhibition of calcium-dependent potassium channels [42], and regulate the mucosal immune function [43]. The brain has impact on gut microbiota through the modulation of the gastrointestinal motility, the mucus production, as well as the alteration of intestinal permeability and immune function [44].

Various preclinical studies implicated a connection between the gut microbiota and the central nervous system [45,46]. Neufeld et al., observed a reduced anxiety-like behavior of germ-free mice accompanied by neurochemical changes in the brain [47], whereas other studies demonstrated an adoptive transfer of behavioral phenotype in germ-free mice via fecal microbiota transfer (FMT), as well as concomitant changes in brain chemistry [48].

Alterations in the gut-brain axis are discussed in the pathogenesis of different psychiatric and depressive disorders, including autism spectrum disorders (ASD) or affective disorders [49], but they are also associated with neurological disorders, like multiple sclerosis (MS), Parkinson’s disease, fibromyalgia [50], or chronic pain syndrome [36]. Even irritable bowel syndrome [51] or obesity [52] are suggested to be linked to an altered gut-brain axis.

The role of gut microbiota in the pathogenesis of these diseases is supported by the observation of an altered gut flora in neurological and mental disorders. For ASD, microbial changes were observed in both clinical and preclinical animal studies, with minor occurrence of *Bacteroides* and an increased abundance of *Clostridium* spp [53,54]. Jiang et al., observed an altered fecal microbiota composition in patients with major depressive disorders with a significant higher abundance of the bacterial phyla Bacteroidetes, Proteobacteria, and Actinobacteria, and significant reduced abundance of Fimicutes in comparison to healthy controls. On the family and genus level, patients with depression showed a significantly increased level of Enterobacteriaceae and *Alistipes* and a reduced level of *Faecalibacterium* [55]. Patients with irritable bowel syndrome showed an increase of Firmicutes and a reduction of Bacteroidetes [56], and some data indicate increased levels of pro-inflammatory bacterial strains (e.g., Enterobacteriaceae) and reduced levels of beneficial bacterial strains, like *Lactobacillus* and *Bifidobacterium* [57,58]. Differential microbiota profiles were also observed in patients with Alzheimer’s disease [59], Parkinson’s disease [60], or MS [61].

Even though a dysbiosis is observed in many neurological diseases, a causative role of the gut microbiota in the pathogenesis remains unclear. However, a normalization of the dysbiosis through the supplementation of probiotics, showed promising effects on clinical neurologic or mental symptoms. Animal studies indicated a reduction of different neurologic symptoms including anxiety, depression or stress after treatment with *Bifidobacterium* and *Lactobacillus,* as well as a modulating effect on neurotransmitter concentrations in the brain [62,63]. Even in human studies, the supplementation of probiotics showed some beneficial effect on anxiety, depression-related behavior, and stress [64,65].

## 6. Microbiota in Health and Disease

Formerly three distinct human enterotypes has been described [66], but follow-up studies have specified only two clusters dominated by *Bacteroides* and *Prevotella*, both belonging to phylum Bacteroidetes [67]. Enterotypes are associated with diets, with *Bacteroides* being predominant in high-protein and high-fat animal diet and *Prevotella* being increased in carbohydrate-rich diet. Interestingly, the bacterial composition changes within 24 h but the enterotypes remain stable during a 10-day diet [67]. In accordance to Wu et al. [67], our own data showed variations after dietary changes (low fermentable carbohydrates, gluten-free diet), but we also noticed a substantial conservation of the individual microbiota. Interestingly, the dietary effect on the bacterial genus level was more pronounced in patients with non-celiac glutensensitivity when compared to healthy controls, thus suggesting a more susceptible and vulnerable microbial composition in these patients [14]. In addition to inflammatory bowel diseases, an altered composition of gut microbiota has been found in various immune-triggered inflammatory diseases, including MS, rheumatoid arthritis (RA), systemic lupus erythematosus (SLE), and psoriasis [18].

### 6.1. Inflammatory Bowel Disease

Patients with inflammatory bowel diseases often show a dysbiosis with an increased ratio of Firmicutes to Bacteroidetes, although it is unclear whether the dysbiosis is crucial for pathogenesis or rather adaptation to persistent inflammation in the gut [68,69,70,71]. Several studies have suggested that bacteria not only trigger the immune system, but inflammation itself can also have a major impact on gut microbiota composition [72,73]. A deranged immune defense that is caused by pathogens or an inadequate immune reaction of the host may also affect the whole immunological situation. Studies have shown that patients with Cohn’s disease have minor bacterial diversity but an increased proportion of Enterobacteriaceae [74]. Furthermore, *Faecalibacterium* und *Roseburia* are diminished in patients with inflammatory bowel diseases. Both organisms are important producers of short-chain fatty acids, such as butyrate, which is regarded as the main fuel for enterocytes and as anti-inflammatory substrate in host defense [75].

### 6.2. Multiple Sclerosis

Multiple Sclerosis is a chronic inflammatory disease of the central nervous system and it is raised by genetic and environmental factors. The pathologic activation of B and T lymphocytes makes them autoaggressive and it leads to an attack on the white matter of the brain. Evidence for the involvement of gut bacteria in MS is underscored by experimental autoimmune encephalomyelitis (EAE), an animal model of MS. The treatment of mice with oral antibiotics caused reduced gut bacteria populations and it resulted in delayed onset and severity of EAE. A shift from a pro-inflammatory Th1/Th17 to an anti-inflammatory Th2 cytokine expression was noticed. Protection came along with increased numbers of IL-10 producing Foxp3+ regulatory T cells, which were suggested to maintain peripheral immune homeostasis [18,76]. Recent studies with fecal samples from Japanese patients with relapsing-remitting MS have revealed a moderate dysbiosis with significantly reduced levels of *Clostridia clusters XIVa* and IV and Bacteroidetes. However, no association between these microbiota and numbers of regulatory T cells has been noticed [77]. The identification of disease-specific microorganisms or bacterial components, which are responsible for the increased susceptibility in MS, will open new therapeutic options [78,79].

### 6.3. Rheumatoid Arthritis

Rheumatoid Arthritis is suggested that the activation of the host immune system by gut microorganisms is involved in RA, a chronic inflammatory disorder. Animal studies showed a 100% incidence of germ-free rats to develop adjuvant-induced arthritis after intradermal injection of *Mycobacterium bovis* or peptidoglycan from *Staphylococcus epidermidis*, while conventional rats were largely protected. It is therefore assumed that commensal gut bacteria are not necessary to develop adjuvant-induced arthritis. In contrast, they are more likely to modulate the immune system and generate a suppressive effect on the development of adjuvant-induced arthritis, which is possibly caused by more dominant suppressor T cells [80]. Animal studies demonstrated that the colonization of germ-free mice with segmented filamentous bacteria caused an imbalance between regulatory and inflammatory T cell response with the expansion of inflammatory Th17 cells [81]. In addition, the stimulation of TLR4 by bacterial lipopolysaccharides resulted in more severe autoimmune arthritis and increased production of inflammatory cytokines in synovial tissue from mice [82]. Contrary results were obtained from studies with rats that demonstrated a decreased activity of adjuvant-induced arthritis after colonization with Gram-negative bacteria, like *E. coli* and *Bacteroides,* possibly via their lipopolysaccharides, while Gram-positive bacteria, like *Bifidobacterium*, *Propionibacterium acnes*, and *Lactobacillus,* were more likely to amplify the disease, possibly via peptidoglycans [83]. A dysbiosis in human RA was already described several decades ago. In 1965, Mansson et al., noticed an increase of *Clostridium perfringens* in stool samples from patients with RA, but later it became clear that many other bacteria were involved in the development of RA [84]. The finding of antibodies to enterobacterial common antigens in human serum and joint fluid samples supports the hypothesis that Enterobacteriaceae are involved in the pathogenesis of RA [85]. Fecal samples from patients with RA further revealed less *Bifidobacteria* and members of *Bacteroides, Porphyromonas*, *Prevotella* group, *B. fragilis* subgroup, and *Eubacterium rectale*, *Clostridium coccoides* as compared to patients with fibromyalgia [86]. Although all data indicate an involvement of gut microbiota in the pathogenesis of RA, further studies are needed to identify the effects of gut microorganisms on RA.

### 6.4. Systemic Lupus Erythematosus

Systemic lupus erythematosus is a systemic autoimmune disease that involves skin, joints, blood cells, heart, kidneys, or lungs, and most commonly SLE patients possess anti-nuclear antibodies or autoantibodies against double-stranded DNA. The severity of symptoms varies widely from patient to patient and women, in particular, are affected by SLE. In addition to genetic or environmental factors, e.g., vitamin D deficiency, a bacterial dysbiosis is also considered to be a triggering factor. Recently, a significantly lower Firmicutes/Bacteroidetes ratio has been found in SLE patients than in healthy controls. The association between Bacteroidetes and SLE was confirmed with significant differences of Bacteroidia (phylum Bacteroidetes) and Clostridia (phylum Firmicutes) between SLE patients and healthy individuals. The taxonomic families Lachnospiraceae and Ruminococcaceae (both phylum Firmicutes) were positively related with healthy controls [18,87]. However, contrasting results were described in a murine lupus model by Zhang et al., Female mice with highest risk of lupus showed an increased level of Lachnospiraceae and Clostridiaceae and a depletion of *Lactobacilli* in gut during disease progression when compared to controls. The feeding of lupus mice with retinoic acid led to the restoration of *Lactobacilli*, which was accompanied by a significant improvement in the symptoms in this murine lupus model [88]. Thus, interaction between gut microorganisms and immune cells seems to be crucial in maintaining tolerance toward self-antigens. Future research must clarify whether dysbiosis is causally involved in SLE or whether an inappropriate immune response leads to a shift in gut microbiota [89].

### 6.5. Psoriasis

Psoriasis is an immune mediated inflammatory disease with the hyperproliferation of keratinocytes. Chang et al. described a significant different skin microbiota pattern with higher diversity and heterogeneity but reduced stability in patients with psoriasis when compared to healthy controls. Increased numbers of *Staphylococcus aureus* were associated with psoriasis, while *S. epidermidis* and *P. acnes* were reduced. Studies with germ-free newborn mice demonstrated that skin colonization with *S. aureus* causes an inflammation by Th17 axis, and it is suggested that the loss of community stability may favor the accumulation of pathogens [90]. Similar results were obtained when analyzing human skin biopsies. *Staphylococci* and *Propionibacteria* were significantly decreased in psoriasis limb skin when compared to controls, whereas Proteobacteria were enriched in the trunk skin of psoriasis patients versus controls [91]. In addition to skin bacteria, intestinal bacteria are also associated with psoriasis. A dysbiosis with minor phylum Firmicutes but enriched phylum Bacteroidetes was described in gut microbiota of psoriasis patients versus controls, and microbiota also differed between patients with severe or mild disease state. Bacteroidia showed higher abundance and *Bifidobacterium* was reduced in severe psoriasis [92]. Others showed lower abundance of *Akkermansia muciniphila* [93], and a lower content of *Faecalibacterium prausnitzii,* but increased *E. coli* in psoriasis, and thus provided further evidence for a disturbed gut-microbiota-skin axis in psoriasis [94].

### 6.6. Obesity

The influence of intestinal bacteria on the outcome of obesity has been described. Mouse studies demonstrated that the transfer of human gut bacteria from an obese twin caused abnormal weight gain in thitherto germ-free housed mice, while intestinal bacteria from the lean twin protected from overweight and obesity-associated metabolic phenotype, although mice received the same low-fat, high-fiber diet [95]. Overweight individuals show an increased ratio of Firmicutes to Bacteroides as compared to normal-weight individuals. Bacteria belonging to phylum Firmicutes are often able to digest long-chain carbohydrates and thus provide the host with additional sources of nutrients. This can lead to increased energy uptake by the host and thus to an inappropriate weight gain and obesity [96,97]. Obesity is further associated with reduced bacterial diversity and altered metabolic pathways [10].

## 7. Probiotics and Prebiotics

Since a well-balanced microbial pattern seems to protect against disease, many efforts have been done to identify beneficial bacteria. In this context, probiotics and prebiotics have gained much attention. Probiotics are defined as living organisms with beneficial effect on health. Most probiotic products contain high concentrations of *Lactobacillus* or *Bifidobacterium* spp., which support the host’s immune system, stimulate the host’s defense through increased production of anti-microbial defensins, regulate gut permeability, and are often the main producers of metabolites, such as vitamins [98]. A recent meta analysis showed a positive effect of probiotics, and especially combinations thereof, in children with inflammatory bowel disease. Probiotics also yielded a promising outcome in adults with ulcerative colitis, but had only minor effects in adult patients with Crohn’s disease [99].

The combination of probiotics and prebiotics could make it easier for probiotics to settle and multiply and may reduce the concentrations of pathogens and undesirable metabolites. Prebiotics are fermentable oligosaccharides, e.g., inulin, oligofructose, or fructo-oligosaccharides that stimulate the growth of beneficial bacteria, such as *Bifidobacterium*. In addition to this bifidogenic effect, some prebiotics (like fructans or arabinoxylan-oligosaccharides) have a major impact on butyrate production, a valuable component that serves as an energy source for enterocytes and contributes to the maintenance of the intestinal barrier [98,99]. Mouse studies have shown that feeding with dietary fibers rich in soluble galacto- and fructo-oligosaccharides changes the microbiota pattern, yields defense against invasive bacteria, and has a positive effect on body weight and glucose tolerance [100].

However, the excessive consumption of these fermentable oligosaccharides is accompanied by increased gas production with flatulence, and a diet low in fermentable oligosaccharides has been shown to improve gastrointestinal symptoms (abdominal pain, bloating, flatulence) in patients with irritable bowel syndrome and patients with non-celiac glutensensitivity [14,101]. The dietary reduction of these oligosaccharides results in minor amounts of Bifidobacteriacea [102,103,104]. However, as mentioned above, *Bifidobacterium* spp. are considered as valuable probiotics in gastrointestinal disorders [105], thus yielding this paradoxical situation that questions the probiotic effect of *Bifidobacterium* spp. In this context, a recent study has shown that probiotic supplementation of patients suffering from irritable bowel syndrome failed in the improvement of clinical symptoms [101]. Other data indicate that the supply with probiotics has no strong influence on microbiota, but rather changes the metabolic activity of resident bacteria [106]. This data underline the complexity of gut microbiota and their interaction with the host and challenge the conventional interpretations and classification of selected bacteria species as beneficial or unfavorable organisms.

## 8. Summary

The colonization of humans with microorganisms begins directly at birth and is characterized by a permanent adaptation to varying conditions. Several factors influence the bacterial composition, and especially the gastrointestinal tract is confronted with many antigens, commensals, and potentially pathogenic microorganisms. The gut associated lymphatic tissue fulfils the important task of exercising tolerance to non-invasive, harmless microorganisms on the one hand and efficiently combating pathogens on the other. Gut microbiota show a very individual composition and the interaction between microbiota and host epithelium, as well as the stimulation of the native and adaptive immune system is the prerequisite for gut homeostasis. A disbalance with over- or under-represented microorganisms is present in several diseases. However, a direct causal role of dysbiosis in the pathogenesis of the diseases often remains unclear and it requires more detailed taxonomic data and functional analyses. In order to properly understand the functional role of intestinal microbiota in gut health, an integrated approach must be adopted that includes all major microbial components (bacteria, viruses, and fungi) that are present in the gut.
